# Critical care specialist nurses’ psychological experience during training in China: a descriptive qualitative study

**DOI:** 10.3389/fmed.2025.1730590

**Published:** 2026-01-20

**Authors:** Yanting Zhang, Jing Ma, Xinbo Ding, Lan Deng, Anlong Zheng

**Affiliations:** Department of Critical Care Medicine, Hubei Clinical Research Center for Critical Care Medicine, Zhongnan Hospital of Wuhan University, Wuhan, China

**Keywords:** critical care, psychological experience, qualitative research, specialist nurses, training experience

## Abstract

**Aim:**

To explore the psychological experiences and feelings of Chinese nurses undergoing critical care specialty training, providing a reference for optimizing training models and guiding professional development.

**Methods:**

A descriptive qualitative design was adopted. A total of 20 nurses who completed critical care specialty training were selected using a maximum variation purposive sampling method (to ensure diversity in age, hospital grade, and department). Data were collected through 15 one-on-one semi-structured in-depth interviews and 1 focus group interview (5 participants). The focus group was used for data triangulation to confirm consistency with individual interview findings. Each interview lasted 30–45 min. Interview data were transcribed verbatim and analyzed using Colaizzi’s 7-step phenomenological method (the sole analytical framework) with NVivo 11 Plus for coding assistance.

**Results:**

Three categories of psychological experiences with nine themes were identified: positive experiences (4 themes): perceived improvement in professional theoretical knowledge; enhanced clinical thinking abilities; expanded career planning; and experience of a comfortable humanistic environment. Negative experiences (2 themes): mismatch between training content/methods and personal needs; high psychological stress from assessments, leadership expectations, and peer competition. Mixed (ambivalent) experiences (3 themes): crowded training venues but well-equipped facilities; incomplete investment of effort during training but high satisfaction with personal performance; non-proactive participation in training but no resistance to it.

**Conclusion:**

Chinese critical care specialty training helps nurses perceive improvements in professional competence and career prospects, but is accompanied by unmet individual needs and psychological stress. Training organizers should design individualized curricula, increase clinical practice opportunities, and provide targeted psychological support to optimize training effectiveness.

## 1 Introduction

In China, specialist nurses are defined as expert clinical nurses with higher nursing education, specialized training, rich clinical experience, and expertise in a specific nursing domain (1). Internationally, equivalent roles include U.S. Clinical Nurse Specialists (CNSs)–advanced practice registered nurses (APRNs) who optimize care for critically ill patients through coordinated, specialized interventions (2). With the global emphasis on improving critical care quality, China has expanded critical care specialist nurse training to enhance Intensive Care Unit (ICU) nursing competence. However, critical care nurses face inherent high stress (3), and qualitative studies on other specialist nurse groups (e.g., oncology, pediatric nurses) have shown that trainees often experience anxiety and psychological distress during training. Psychological state directly affects participation in training, knowledge transformation efficiency, and career retention rate. The high-pressure nature of critical care nursing makes the impact of trainees’ psychological experiences on training effectiveness even more crucial (3).

Despite the global growth of specialized nursing education, research on the psychological experiences of critical care nurse trainees remains limited, particularly in non-Western contexts. International studies have highlighted stress from clinical skill demands and career uncertainty (4), but few have explored how system-specific factors (e.g., China’s decentralized training curricula) shape psychological experiences. In China, training for specialist nurses lacks unified standards, with content and methods determined by individual institutions (5). This inconsistency may lead to unmet trainee needs, yet no studies have systematically analyzed how these gaps affect psychological well-being or training engagement. Therefore, although the training of critical care specialist nurses has been widely carried out in China, there is still a lack of qualitative research on trainees’ subjective psychological experiences. Moreover, existing international studies mostly focus on Western contexts and lack exploration of psychological experiences under specific conditions such as China’s decentralized training system and collectivist culture.

This study aimed to explore the psychological experiences of Chinese nurses undergoing critical care specialty training, using adult learning theory and the stress-coping model. Adult Learning Theory was used to guide the research design and focus on trainees’ needs, while the Stress-Coping Model was applied to analyze the emergence and regulation of psychological stress, providing a theoretical foundation for the discussion. Findings seek to inform the optimization of global critical care training programs, particularly in contexts with decentralized curricula.

## 2 Materials and methods

### 2.1 Study design

A descriptive phenomenological design was adopted, focused on capturing the “psychological experience” of critical care specialist nurses during training. This framework was chosen for its ability to explore subjective psychological perceptions, aligning with the study’s aim to understand trainees’ feelings and needs. Colaizzi’s 7-step method was used for analysis: (1) reading original materials; (2) extracting significant statements; (3) coding statements; (4) categorizing codes; (5) describing the phenomenon; (6) constructing a thematic framework; (7) member checking (6, 7).

### 2.2 Setting

The study was conducted at a national-level critical care training base (a tertiary teaching hospital in Wuhan, China) with a 10-years history of training specialist nurses. The 8-weeks training program combined online (20%, pre-recorded theoretical lectures) and offline (80%, clinical practice and in-person workshops) components. For more information about this training course (80%, clinical practice and in-person workshops), please refer to Supplementary Material 1. Evaluations included:

- Theoretical exam (100 points, ≥80 points to pass; >95 = excellent, 85–94 = good, 80–84 = pass);
- Clinical skills assessment (100 points, same scoring as theory);
- Scenario simulation teaching/lecturing/case report (100 points, ≥60 to pass; >85 = excellent, 75–84 = good, 60–74 = pass).

Graduation requirement: all assessments must meet or exceed the passing score.

The research team adopted the “phenomenological epoche” strategy to sort out their own critical care training experiences and potential biases (such as assuming that “training should enhance professional identity”) before data collection. They also recorded reflective journals through regular team discussions to avoid bias influencing data interpretation.

### 2.3 Participants

#### 2.3.1 Sampling strategy

Maximum variation purposive sampling was used to ensure diversity: a stratified sampling method was employed according to four dimensions: “age (25–30 years old, 31–40 years old, over 40 years old), hospital level (tertiary/secondary), ICU type (general ICU/specialized ICU), and professional title (junior/intermediate).” At least three participants were included for each dimension, and ultimately 20 samples were determined. Recruitment occurred from April to June 2025, with participants selected if they met the following criteria:

1. Held a valid Nurse Practice Certificate of the People’s Republic of China;
2. Completed ≥1.5 months (6 weeks) of critical care training (consistent with the 8-weeks program, allowing for 2 weeks of potential absences);
3. Were interviewed within ≤2 weeks of training completion (to minimize recall bias);
4. Provided voluntary informed consent.

Exclusion criterion: accumulated leave ≥ 14 days during training (to ensure sufficient exposure to training content).

#### 2.3.2 Sample characteristics

A total of 20 nurses were included (15 in individual interviews, 5 in 1 focus group). Sample characteristics are shown in Table 1, with representation across 10 provinces, 2 hospital grades, and 6 ICU subtypes. Data saturation was achieved after 18 interviews, interviews 19 and 20 were conducted solely to verify existing themes, with no additional information found, thus confirming our sample size was sufficient.

**TABLE 1:** General information of interviewees.

Interview format	Number	Gender	Age	Economic income	Marital status	Professional title	Working time (year)	Education level	Whether a department manager	Hospital grade	Work location	Source department
One-on-one interview	N1	Female	31	>10000	Married	Junior Professional Title	8	Bachelor’s Degree	Yes	Tertiary Hospital	Hubei Province	Cardiothoracic Surgery Intensive Care Unit
	N2	Female	35	8000–10000	Married	Intermediate Professional Title	14	Bachelor’s Degree	Yes	Tertiary Hospital	Guangdong Province	Department of Critical Care Medicine
	N3	Male	33	>10000	Married	Intermediate Professional Title	11	Bachelor’s Degree	Yes	Tertiary Hospital	Zhejiang Province	Department of Critical Care Medicine
	N4	Male	31	>10000	Married	Intermediate Professional Title	10	Bachelor’s Degree	Yes	Tertiary Hospital	Shanxi Province	Surgical Intensive Care Unit
	N5	Female	35	5000–8000	Married	Intermediate Professional Title	13	Bachelor’s Degree	Yes	Secondary Hospital	Guangxi Zhuang Autonomous Region (GZAR)	Department of Critical Care Medicine
	N6	Female	36	8000–10000	Married	Intermediate Professional Title	12	Bachelor’s Degree	No	Tertiary Hospital	Jilin Province	Emergency ICU
	N7	Male	32	>10000	Married	Intermediate Professional Title	11	Bachelor’s Degree	Yes	Tertiary Hospital	Guangxi Zhuang Autonomous Region (GZAR)	Department of Critical Care Medicine
	N8	Female	33	>10000	Married	Intermediate Professional Title	11	Bachelor’s Degree	No	Tertiary Hospital	Shandong Province	Anesthesiology and Critical Care Medicine
	N9	Female	28	>10000	Unmarried	Intermediate Professional Title	6	Bachelor’s Degree	No	Tertiary Hospital	Hainan province	Respiratory Intensive Care Unit
	N10	Female	37	5000–8000	Married	Intermediate Professional Title	14	Bachelor’s Degree	Yes	Tertiary Hospital	Xinjiang Uygur Autonomous Region	Department of Critical Care Medicine
	N11	Female	31	3000–5000	Married	Intermediate Professional Title	9	Bachelor’s Degree	No	Tertiary Hospital	Yunnan Province	Neurosurgical Intensive Care Unit
	N12	Female	31	5000–8000	Married	Intermediate Professional Title	10	Bachelor’s Degree	No	Tertiary Hospital	Shanxi Province	Department of Critical Care Medicine
	N13	Male	31	>10000	Married	Intermediate Professional Title	11	Bachelor’s Degree	Yes	Tertiary Hospital	Tianjin	Department of Critical Care Medicine
	N14	Male	30	3000–5000	Married	Junior Professional Title	7	Bachelor’s Degree	No	Secondary Hospital	Henan Province	Department of Critical Care Medicine
	N15	Female	34	8000–10000	Unmarried	Junior Professional Title	13	Associate Degree	Yes	Tertiary Hospital	Fujian Province	Neurosurgical Intensive Care Unit
Focus group interview	G1	Female	38	>10000	Married	Intermediate Professional Title	18	Bachelor’s Degree	Yes	Tertiary Hospital	Gansu Province	Department of Critical Care Medicine
	G2	Female	32	5000–8000	Unmarried	Junior Professional Title	10	Associate Degree	Yes	Tertiary Hospital	Fujian Province	Neurosurgical Intensive Care Unit
	G3	Female	35	5000–8000	Married	Intermediate Professional Title	14	Bachelor’s Degree	Yes	Tertiary Hospital	Jiangxi Province	Department of Critical Care Medicine
	G4	Female	34	>10000	Married	Intermediate Professional Title	10	Bachelor’s Degree	Yes	Tertiary Hospital	Hubei Province	Department of Critical Care Medicine
	G5	Female	34	8000–10000	Married	Intermediate Professional Title	13	Bachelor’s Degree	No	Tertiary Hospital	Zhejiang Province	Emergency ICU

This study will adopt a strategy of “giving priority to individual interview contents and supplementing with focus group interviews”: first, independently analyze the individual interview data to form preliminary themes, and then verify theme consistency through focus group data (for example, the theme of “mismatch between training content and needs” appears frequently in both sets of data). Meanwhile, the focus group data supplements collective experiences not covered in individual interviews (such as “group resonance of peer competition pressure”), ultimately integrating to form a unified thematic framework.

### 2.4 Data collection

#### 2.4.1 Tools

Semi-structured interview guide: Developed based on adult learning theory and pilot-tested with 2 nurses (not included in analysis) to refine clarity. Core open-ended questions included:

1. “Can you share your overall feelings about this critical care training?”
2. “What aspects of the training content or methods were most/least helpful to you?”
3. “Did you experience any stress during training? If so, what contributed to it?”

[Full guide in Table 2, with redundant questions (e.g., duplicate basic information) removed.]

**TABLE 2:** Semistructured interview guide.

Semistructured interview questions
(1) How long have you been working as a clinical nurse?
(2) What is your purpose for attending this training?
(3) Could you share your experience with the environment of this clinical training venue?
(4) How do you feel about the level of instruction in this training?
(5) What do you think about the content or format of the clinical practice in this training?
(6) Is the content of this training practical? Can you apply the knowledge learned to your clinical work?
(7) Did you feel anxious or under great psychological pressure during the training process? (If so, what was it from? If so, at what stage? How did you decompress? When did it ease? How much were you fully engaged in this training?)
(8) Do you feel that your various clinical abilities (such as professional knowledge, clinical skills) have greatly improved compared to before through this training?
(9) Do you think this training has helped your future career planning? Can you share specific aspects?
(10) What do you think are the shortcomings of this training?
(11) Do you have any comments or suggestions for this training?
(12) Is there anything I should have asked but did not?
(13) Is there anything else you would like to add before we end the interview?

Demographic questionnaire: Collected gender, age, income, professional title, work experience, education level, hospital grade, and department, marital status and number of children.

#### 2.4.2 Procedures

Interviews were conducted within 1 week post-training (to ensure memory accuracy) in a private, undisturbed hospital meeting room.

The interviewer (a PhD-prepared nursing researcher with 5 years of qualitative experience, independent of the training program) introduced the study purpose, emphasized voluntary participation, and guaranteed anonymity (participants were assigned IDs like N1, G1).

Interviews were audio-recorded and transcribed verbatim within 24 h. Field notes captured non-verbal cues (e.g., hesitation, emotional tone).

Focus group interview: Moderated by the same researcher, with a semi-structured guide to explore shared experiences (e.g., “How did you all perceive the balance between theory and practice?”). Group size (5 participants) was chosen to encourage open discussion.

### 2.5 Quality control

Rigor: Ensured through triangulation (individual + focus group interviews), member checking (10 participants reviewed transcripts/themes for accuracy), peer debriefing (2 qualitative researchers independently coded 20% of data, achieving 85% inter-coder agreement), and an audit trail (documenting all analytical decisions).

Ethics: Approved by the Medical Ethics Committee of Zhongnan Hospital of Wuhan University (No. 2024112K, May 15, 2024). Participants signed written informed consent, with the right to withdraw at any time without penalty. To mitigate coercion risks (many participants were sent by their hospitals), the researcher clarified that participation would not affect training evaluation, and interviews were conducted separately from training staff.

### 2.6 Data analysis

**FIGURE 1:**
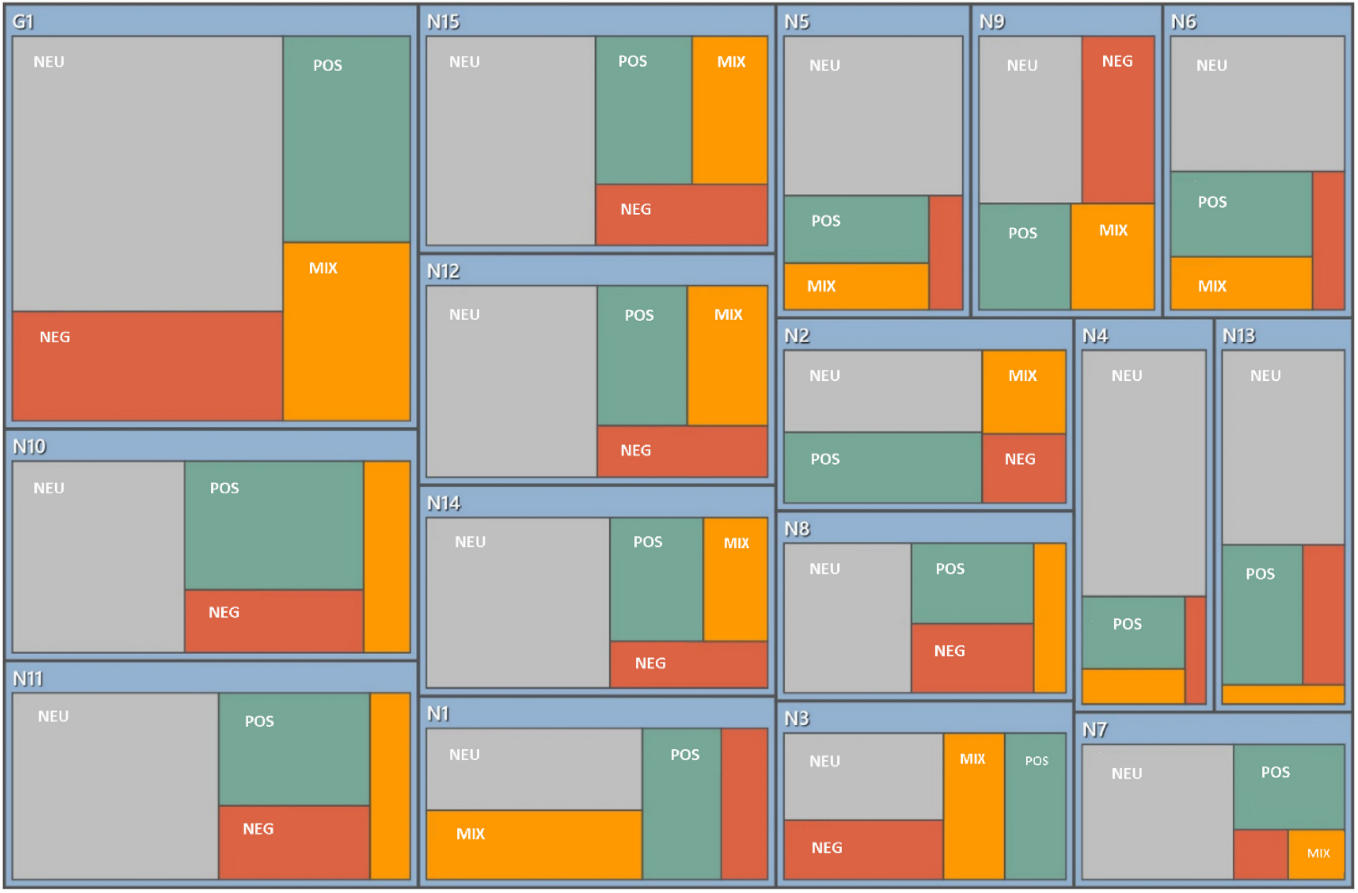
Emotion coding diagram. NEU, neutrality; POS, positive; NEG, negative; MIX, mixed. The percentages for emotional coding are purely descriptive and do not involve quantitative analysis.

NVivo 11 Plus was used for coding management, but Colaizzi’s 7-step method was the sole analytical framework (grounded theory terminology like “axial coding” was removed to avoid methodological confusion) (8–11). Steps included: ➀ Reading all transcripts to immerse in the data; ➁ Extracting significant statements (e.g., N1: “Through training, my knowledge reserves have expanded”); ➂ Assigning codes to statements (e.g., “theoretical knowledge improvement”); ➃ Categorizing codes into subthemes (e.g., “positive experiences”); ➄ Describing the phenomenon (e.g., “trainees reported enhanced confidence in clinical decision-making”); ➅ Constructing a thematic framework (Figure 1); ➆ Validating with participants and peer researchers.

Emotional coding serves as a descriptive auxiliary tool only, aiming at intuitively presenting the distribution of trainees’ emotional tendencies without conducting quantitative statistical analysis. The coding is based on emotional vocabulary (such as “anxious,” “satisfied”), tone descriptions (such as “hesitant,” “firm”), and contextual clues in interview texts, with two researchers independently performing the coding. Its limitation lies in possibly failing to capture dynamic changes in emotions.

## 3 Results

### 3.1 Interviewee’s general information

In this study, the sample size adhered to the principle of “information saturation,” incorporating nurses of different ages and regions who participated in critical care specialty course training, thereby enhancing the representativeness of the sample size. A total of 20 samples were included in the final analysis. Among them, 15 were included in one-on-one interviews, and 5 were included in focus group interviews. General information about the interviewees can be found in Table 1.

### 3.2 Emotion coding diagram

The NVivo sentiment coding diagram (Figure 1) showed: 35% neutral, 30% positive, 25% negative, and 10% mixed emotions. “Neutral” was defined as a lack of strong emotional valence (e.g., objective descriptions of venue conditions), while “mixed” reflected conflicting feelings (e.g., satisfaction with performance despite incomplete effort). In this study, we have merged “neutral theme” and “mixed theme” into “mixed theme,” which refers to psychological states that simultaneously contain both positive and negative elements.

### 3.3 Interview results

After repeatedly comparing, summarizing, and refining the interview data from 20 respondents, three categories of psychological experience themes were identified. These include 4 positive psychological experience themes: perceived improvement in professional theoretical knowledge, enhanced clinical thinking abilities, expanded career planning, and comfortable humanistic environment. 2 negative psychological experience themes: mismatch between training content/methods and personal needs, and High psychological stress. And 3 mixed psychological experience themes: crowded venues but well-equipped facilities, incomplete effort but high performance satisfaction, and non-proactive participation but no resistance.

#### 3.3.1 Positive psychological experience

**(1) Theme 1: Perceived improvement in professional theoretical knowledge**

Trainees reported enhanced understanding of ICU-specific topics (e.g., hemodynamics, ventilation).

*N1: “Through the training, there is undoubtedly an increase in my own knowledge reserves and expansion.”*

*N5: “Professional knowledge has improved compared to before because it all pertains to critical care. Whether it is theoretical knowledge or some techniques, I feel like I have stepped up a level.”*

*N11: “Throughout the entire training period until the end, whether it is my overall concept of patient care or the attention to detail in patient care, I think the improvement is quite significant and extensive for me.”*

Second, respondents believe that the course design for ICU specialist nurse training is reasonable and engaging, which can stimulate interest in learning.

*N13: “The arrangement of the work visit allows everyone to engage in hands-on practice. The course format is quite vivid, and the scheduled rounds provide a unified approach with the teachers’ guidance on processes, offering us a guiding principle.”*

*N14: “Through a month of online learning, as well as clinical practice, including rounds, workshops, and internships, such a course setup is very beneficial for me.”*

*N11: “I truly like the teaching method of scenario simulation. In a simulated situation, I can personally experience it from various perspectives, such as a participant, observer, or stakeholder, which significantly helps me understand what aspects I need to consider more when I do this in clinical practice.”*

**(2) Theme 2: Enhanced clinical thinking abilities**

Training shifted trainees from “task-oriented” to “evidence-based” thinking.

*N1: “I feel that the nursing mindset in a comprehensive ICU differs from that in a specialized ICU. In a comprehensive ICU, there is more emphasis on holism, systematic approaches, and focus. This has led to some changes in my overall thinking about patient care after returning.”*

*N2: “In terms of research, the training has at least given me a basic framework. After returning, I know where to start; for example, evidence-based practice requires solid evidence to determine nursing diagnoses and interventions.”*

**(3) Theme 3: Expanded career planning**

The interviewees stated that specialist nurse training has been beneficial to their career development, broadening the path for their specialization. It has also made their future work plans, goals, priorities, and professional positioning clearer, instilling confidence in their job prospects, enhancing their professional attitudes, and fostering a willingness to proactively advance their careers. Such as specializing in CRRT or moving into management.

*N1: “Although I’m not very proficient in critical care ultrasonography at the moment, I now know the direction I want to take in the future.”*

*N3: “In the short term, I plan to share some of the things I have learned here first and then improve my Continuous Renal Replacement Therapy (CRRT) management because there’s still a lot of quality control work to be done on my end. I believe that studying here can make our related management more standardized.”*

*N5: “Since our department is technically quite advanced, what we need more of is now various types of training, such as different training models, because we might have always followed the same old pattern. However, now, I feel like I have learned many different teaching methods. Currently, I aim to excel in the role of head nurse, maintaining a learning attitude and leading by example.”*

**(4) Theme 4: Comfortable humanistic environment**

The training site’s low-noise culture and mentor support were valued.

*N1: “During rounds, 20–30 people spoke softly, keeping noise* < *60 decibels–this calm environment helped me focus.”*

*N5: “One thing I particularly like here is the great working atmosphere among everyone, as well as the rich cultural atmosphere of the department.”*

*N9: “Each of us had a mentor who supported our academic and personal needs–this care made me feel valued.”*

#### 3.3.2 Negative psychological experience

**(1) Theme 1: Mismatch between training content/methods and personal needs**

The interviewee stated that, owing to varying individual needs, the acceptance of certain training content was not very high. This includes:

➀ Theoretical content was either too advanced or irrelevant:

*N3: “Some trainees questioned why they needed to learn electrocardiograms if their hospitals don’t use them.”*

➁ Insufficient clinical practice:

*N2: “We follow doctors on rounds for 1 h/day–we need more hands-on time, like practicing nasoenteric tube placement.”*

*N3: “The whole morning is focused on lectures, which I personally feel is a bit of a waste of time. For example, after entering the clinic, I put on a mask, a hat, stay for an hour, and then I leave. I think the time for clinical practice is too short, and it is not a continuous learning process, which makes me feel somewhat disconnected.”*

➂ Poor online learning effectiveness:

*N12: “I feel the online sessions are somewhat inferior because it appeared to be some participants finished listening to all the lectures soon after they started. I do not know how they managed, but I would wake up around seven or eight in the morning, take a break for lunch and rest, then sit through until the afternoon. I was constantly attending lectures every day, but I felt the results were not as good. I think in-person sessions would be better.”*

*N15: “Night shift participants only logged into online lectures without listening; in-person sessions are much better.”*

*G1: “Regarding online courses, I think some teachers deliver excellent lectures that are closely related to clinical practice and very practical. However, there are others I cannot stand to listen to; they seem useless, and without immediate feedback, I lose interest.”*

**(2) Theme 2: High psychological stress**

➀ During the training process, due to concentrated theoretical knowledge training, weak foundations and high assessment pressure exist.

*N1: “After the theoretical exam schedule was announced, I started feeling pressure. I thought about it and realized that the exam would take place the day after reporting. Although I had engaged in online learning, the time felt too tight, and I was still very anxious.”*

*N14: “Initially, there might be a bit of pressure because there is so much theoretical knowledge that cannot be fully understood in a short time. I feel like I might not be able to complete this task, which is somewhat stressful.”*

➁ Fewer research courses are offered, and the duration is short, resulting in high pressure to write and report case studies.

*N10: “The pressure comes when working on case studies because you have to review many literatures, which I rarely did before. My literature review skills are relatively weak.”*

*N11: “The pressure was quite high before presenting the case study. It was my first time dealing with this, and I felt like I knew nothing. I did not know how to proceed with writing the case study or how to present it. Other teachers might be more familiar with this, so I felt a lot of pressure.”*

➂ Peer participants are strong, and themselves are relatively weak, leading to significant psychological stress from peers.

*N2: “I am most anxious when writing and presenting case studies because I am not young anymore, and I have been working for many years. Unlike some of them who were from 1995 to 1996, I am considered older. Having worked for so many years, I do not say I do the best, but at least I can say I am worthy of myself and can pass my own standards. Therefore, I try my hardest to do the best, but sometimes my abilities are just there.”*

*N11: “When working on case reports, seeing other participants doing it effortlessly while I am still organizing various materials, and they have already moved on to the next step, makes me feel under a lot of pressure.”*

➃ High expectations from one’s own hospital leadership lead to concerns about insufficient capabilities and not meeting leadership demands.

*N1: “Coming here for training costs money. Although it is not out of my pocket, it is still a significant training fee. Therefore, at that time, I was worried that if I failed the exam, having been given this opportunity by the hospital and not managing well would be unacceptable.”*

*N2: “The leadership puts a lot of pressure on me. He would tell me what to look at, what to learn, like the nasoenteric tube placement technique. I told my leader that I could only try my best to learn. I am here for 2 months, not 2 years; I can only learn as much as possible. I told my leader not to have too high expectations for me.”*

*N14: “The pressure from the leadership is quite high. I worry about what to do if I cannot complete the tasks he has given me after returning from this training. It is somewhat stressful psychologically.”*

#### 3.3.3 Mixed psychological experience

**(1) Theme 1: Crowded venues but well-equipped facilities**

Discomfort from limited space coexists with satisfaction from advanced equipment.

*N1: “Perhaps it is influenced by the patient intake volume. In terms of the indoor environment, our place is relatively more spacious because we have only 15 beds, and our area is slightly larger. Additionally, there are too many instruments and equipment here; we do not have as much back home, so it does not feel crowded. However, this is something the department cannot change.”*

*G2, G5: “It would be better if the environment were more spacious, but in terms of hardware facilities, this place is quite strong, with many comprehensive and advanced instruments, equipment, and technologies.”*

**(2) Theme 2: Incomplete effort but high performance satisfaction**

Most trainees rated their effort 7–9/10 (due to work-family conflicts), yet were satisfied with results.

*N1: “I balanced hospital projects and my child’s illness, so I gave 8.5/10–but I’m proud of my performance.”*

*N6: “I believe I deserve at least 8 because, after returning to my residence and having meals, I habitually turned on my computer to replay previous theoretical courses, possibly focusing on topics that interested me for the day and conducting literature searches. However, I did not sit through the entire session. I might have played with my phone in between or been rather casual; if it was something unfamiliar to me, my completion might not have been as good as everyone else’s, but I was earnestly engaged in the task.”*

*N15: “I would score myself a 7, believing that at most, 5 points out of the 7 reflect my effort in case reporting. Although I may not have performed excellently, I genuinely invested myself, researched literature thoroughly, and was very committed. However, I feel that insufficient time and some domestic distractions may have hindered my progress.”*

**(3) Theme 3: Non-proactive participation but no resistance**

Participation was often hospital-mandated, but trainees recognized benefits:

(1) Departmental development needs. *N1: “The main reason for coming here is some requirements for departmental development. Currently, our department has some deficiencies in nursing; for example, we have not started nasogastric tube placement techniques, ultrasound techniques are also immature, and pulmonary rehabilitation implementation is poor, so I wanted to learn about them.” N2: “Since we are a newly established department without specialist talent training, our director hoped I could come out and learn something to bring back, understand some related knowledge to drive the department’s development.” N13: “Participating in critical care specialist nurse training is to come out and learn because now the department is developing, and our leaders mean to learn more advanced experiences from other places, then see if we can apply them in our department after returning, and then send everyone out.”*
(2) Personal development needs. *N3: “I wanted to come out and learn the experience of other hospitals to broaden my horizons, and another thing is to exercise my thinking.” N11: “I wanted to further improve myself, and then I could be relatively more prominent in the department team.” G3, G4: “I came here mainly to enhance my clinical skills because everyone is too excellent.”*

## 4 Discussion

The purpose of this study was to explore the psychological experiences of Chinese nurses during their critical care training. For most nurses who receive training to obtain a specialty nurse certification, assessments during training, leadership expectations, and the “excellence” of peers can be sources of stress, even though they are considered meaningful learning experiences. In China, the cultivation of specialist nurses has become widespread. Research (12) indicates that specialist nurses can effectively reduce the incidence of postoperative complications in patients by providing efficient and professional nursing services, shortening their hospital stay, helping reduce hospitalization costs, and improving their quality of life. Gaining insight into the lived experiences of trainee specialist nurses is essential. This analysis allows us to improve the training process, enhance program quality and effectiveness, and create more efficient training strategies.

### 4.1 ICU specialist nurse training benefits nurses and positive experiences align with adult learning theory

The results of this study show that after receiving specialized training from ICU nurses, participants reported a significant improvement in their theoretical knowledge and clinical practice abilities, laying a foundation for further expansion of their career planning. The reasons for this include, on the one hand, the systematic theoretical training, clinical practice exercises, and professional clinical guidance provided during the training process, which equipped the trained nurses with cutting-edge theoretical knowledge and practical skills. Additionally, their clinical thinking was enhanced, and their nursing philosophy was updated and supplemented. Moreover, diverse forms of training, such as scenario simulations, group workshops, case writing and reporting, and clinical practice, along with a positive humanistic environment, fully stimulate the enthusiasm of learners to learn, which is consistent with the results of other related studies in China (13, 14). As is well known, exemplary nurses can influence the professional development of nursing students (15). Our study also revealed that specialized nurses were positively influenced by this effect. During the specialized nurse training process, nurses come into contact with excellent mentor teachers and communicate and learn with them. The professional nurses’ good management of the complex ICU environment and patient care broadened the trainees’ horizons and inspired them to strive for self-improvement in a professional sense. This also helped them define their own developmental goals and career directions.

Additionally, while some trainees have indicated improvements in their theoretical knowledge and clinical skills, they still feel uncertain about their career development. Some trainees mentioned that their hospitals do not offer clear career paths for specialized nurses, leading to difficulties in applying what they have learned. This may be due to the relatively late start of specialized nursing development in our country, where the role definition and professional impact have not yet been fully established into a mature system, causing confusion among trainees about their career development (16). Therefore, educators and administrators should further assist specialized nurses in clarifying their responsibilities and roles during training and strive to build a system for the application, development, and management of specialized nursing that suits our national conditions at the governmental and societal levels, enabling them to fully realize their professional value.

Trainees’ perceived improvements in knowledge and career planning reflect adult learners’ need for “relevant, goal-oriented education” (17). The humanistic environment (mentor support, low-noise culture) also aligns with adult learning principles, which emphasize psychological safety (18). These findings echo international studies–e.g., David found that positive training environments enhance nurse trainees’ professional identity (18).

### 4.2 Negative experiences highlight systemic gaps

The results of this study show that, owing to varying individual needs, some participants feel that the training content is not fully aligned with their personal requirements. The mismatch between training and needs reflects China’s decentralized curricula. For example, specialized ICU nurses (e.g., respiratory ICU) wanted ventilator-focused content, while managers sought ICU management training–yet the curriculum was one-size-fits-all. This aligns with Mi et al.’s (19) finding that critical care training lacks individualization (20). The training schedule (Supplementary Material 1) shows that Weeks 2–4 dedicate afternoons to “case report writing,” which directly corroborates trainees’ stress about case reports. The first-week theoretical exam also explains early assessment anxiety. This schedule’s rigidity (no flexible modules) further justifies the need for individualized curricula.

China’s training models, the content of theoretical lectures is usually arranged before the arrival of participants, especially in the blended online and offline model. The content for online lectures is typically prerecorded by the instructor and then uploaded to the teaching system for participants to learn at their own pace. This teaching model struggles to consider the individual learning needs of trainees. In previous studies on training for other specialist nurses (19, 21), this significant issue has also been reflected, where the clinical background, educational level, and professional learning experience of participants significantly influence their needs. For example, many participants hope for more opportunities for clinical practice, as the time allocated for theoretical lectures far exceeds that allocated for clinical practice. This is especially true for nurses, who require clinical practice to provide better care for patients, particularly in the ICU, where the time spent interacting with patients and operating various instruments and equipment greatly exceeds that in other specialties. The participants who are managers prefer to learn about management knowledge and models in the ICU. Additionally, some participants mentioned that when they return to their own hospitals, it is difficult to apply some theoretical knowledge clinically, partly because their hospital’s technology is not mature and partly because of a lack of instruments and equipment. Moreover, in our study, the needs of participants from specialized ICUs and general ICUs were not consistent; participants from specialized ICUs hoped to learn courses that are highly relevant to their specialty, such as those from respiratory intensive care units, who wished to further learn about ventilators and pulmonary rehabilitation-related lecture content.

Therefore, it is crucial to develop targeted training programs on the basis of the actual needs of trainees. We suggest that managers and trainers conduct a comprehensive assessment of individual trainee needs before training and tailor the training content to their varying educational levels, clinical positions, developmental needs, and technical skills; at the same time, they should enhance communication and interaction between mentors and trainees, provide more targeted clinical practice opportunities, and strive to meet the learning needs of trainees as much as possible. Additionally, by drawing on advanced and proven training models from abroad, continuously analyzing the causes of existing problems in training and summarizing experiences, we can make ongoing improvements to course design and training methods, thereby achieving the desired training outcomes.

### 4.3 Mixed experiences reflect compromised autonomy

Non-proactive participation (hospital-mandated) but no resistance highlights the tension between institutional demands and personal growth. This is unique to collectivist cultures like China, where nurses often prioritize organizational needs. However, the acceptance of training benefits suggests potential for improving engagement through needs-based curricula.

In this study, the psychological stress experienced by ICU specialist nurses during their training primarily originated from assessments during the training period, leadership expectations, and competition among participants. Most trainees mentioned that their participation in specialist nurse training was due mainly to personal development and the departmental needs of the hospital. If given the choice, they might not necessarily opt for such training. This reflects the prevalence of “being assigned to participate in training by the hospital” under a collectivist culture, and the influence of hierarchical hospital cultures on “pressure from leadership expectations.” On the one hand, there were family commitments; on the other hand, there were expectations and tasks from their own department leaders, with concerns about falling short of expectations, yet they were still willing to undergo such training to further enhance their capabilities. This was confirmed by the emotional coding map of the nurses, which showed that most participants were not averse but rather mixed in their emotional expression. At the beginning of the training, although it was a “selected” decision by the hospital, trainees usually approached specialist nurse training with curiosity and anticipation of benefits (enhancement of personal abilities and recognition of their department), resulting in a positive and exciting mood.

However, as training progressed, with increased assessment pressure, more academic tasks, and expectations and demands from the hospital, doubts about their own abilities emerged, leading to role conflict. Some trainees lack the experience and ability to handle these issues, resulting in a series of negative psychological experiences, which is consistent with the findings of Ding et al. (22). However, further in-depth interviews revealed that these trainees typically experienced relief from pressure after assessments and the completion of academic tasks, leading to feelings of achievement and value. They could alleviate their stress during the training period through various means, such as calling family, listening to music, walking, enjoying food, and sleeping. Generally, no excessive intervention was needed, as they could regulate themselves. The reason might be that nurses who participated in specialist nurse training had mostly been working for over 5 years and possessed certain stress resistance and work experience. Faced with various tasks during training, in addition to initial discomfort, they gradually became capable of self-regulation and planning as they delved deeper into their studies. However, the curriculum for ICU specialist nurse training is extensive and complex, requiring more practical operations than other types of specialist nurse training do, thus making the learning tasks and arrangements more compact. Managers still need to pay attention to the negative psychological experiences of trainees during the training period, address the sources of stress, take necessary measures to promptly help trainees solve practical life and work-study problems, relieve psychological pressure, meet the individual training needs of trainees, enhance training effectiveness, and improve trainee capabilities.

## 5 Limitations

This study has several limitations: single-center, small sample: the study was conducted at one training base, with 20 participants (85% female). Multi-center sampling (including rural hospitals) and larger samples would improve generalizability. Cross-sectional design: interviews post-training could not capture dynamic psychological changes (e.g., stress fluctuations during training). Longitudinal designs are recommended. Potential bias: leading questions (e.g., “Did you feel anxious?”) may have influenced responses–future studies should use more open-ended prompts. Furthermore, we acknowledge that the interviewer’s academic background may have inadvertently prompted participants toward more scholarly expressions and introduced a risk of leading questions. And the emotional coding used has the limitation of potentially failing to capture the dynamic nature of emotions.

## 6 Conclusion

This study explored the psychological experiences of Chinese critical care specialist nurse trainees, identifying positive (knowledge gain, career clarity), negative (needs mismatch, stress), and mixed (ambivalent participation) themes. These findings highlight the need to optimize training through individualized curricula, increased clinical practice, and psychological support. And the findings of this study are applicable to critical care nurse specialist training within similar decentralized training systems and collectivist cultural contexts. Future research should explore how psychological experiences impact training effectiveness and patient outcomes to strengthen the clinical relevance of specialized nursing education.

## Data Availability

The raw data supporting the conclusions of this article will be made available by the authors, without undue reservation.
