# Supplementary material for: Critical care specialist nurses’ psychological experience during training in China: a descriptive qualitative study
Source: Front Med (Lausanne). 2026 Jan 20;12:1730590. doi: 10.3389/fmed.2025.1730590 (PMC12864049; doi:10.3389/fmed.2025.1730590)
Supplement: Supplementary file 1 [file Table_1.docx]

**Critical Care Specialist Nurse Training Program Clinical Practice Plan**

**I. Training Objectives**

1. Master the roles and functions of ICU nurses.

2. Skillfully practice critical care clinical nursing techniques.

3. Proficiently use common ICU monitoring equipment.

4. Learn to analyze clinical data (analysis of common tests or laboratory results).

5. Be familiar with medical rounds and various clinical nursing teaching models.

**II. Training Content and Requirements**

1. During the training period, participants will join doctors on rounds and participate in nursing handovers, follow mentors in daily care of critically ill patients, and complete internship tasks according to the training plan.

2. In the first month, participants are required to review literature and, under the guidance of their teacher, complete case writing. In the second month, they will practice four procedures under the guidance of their mentors and undergo assessment.

3. Each participant must complete a 5-minute PowerPoint presentation during the training period, covering hospital features, nursing rounds, book report meetings, etc.

4. In the fourth week of training, an interim evaluation of teaching and learning will be conducted. In the eighth week, teachers will organize participants to focus on summarizing learning experiences and evaluating teaching and learning, listening to opinions and suggestions.

**III. Training Plan**

**First Phase: ICU Workflow and Roy Model**

| **The First Week** | Monday | Morning | Welcoming Ceremony |
| --- | --- | --- | --- |
|  |  |  | Introduction to the Hospital Environment |
|  |  | Afternoon | Introduction to Three Wards, Allocation of Lockers |
|  | Tuesday | Morning | Following up with doctors on rounds |
|  |  |  | Handover Shift |
|  |  |  | Responsibilities and Procedures for Each Shift |
|  |  | Afternoon | Introduction to Roy's Model |
|  |  |  | Nursing Issues and Nursing Measures |
|  |  |  | Interpretation of Nursing Case Scoring |
|  | Wednesday | Morning | Following up with doctors on rounds |
|  |  |  | Admission Process/Preparation of Bed Units |
|  |  |  | Discharge Process |
|  |  |  | Outpatient Examinations |
|  |  | Afternoon | Sharing of Excellent Case Studies |
|  |  |  | How to Create a Satisfactory PPT |
|  | Thursday | Morning | Following up with doctors on rounds |
|  |  |  | How to Create a Satisfactory PPT |
|  |  | Afternoon | ICU Patients Outpatient Examination Group Discussion |
|  |  |  | ICU Patients Outpatient Examination Group Discussion |
|  |  |  | Sharing of Literature on Transfers |
|  | Friday | Morning | Following up with doctors on rounds |
|  |  |  | Case Study Collection |
|  |  | Afternoon | Literature Search |
|  |  |  | Theoretical Exam |
|  |  |  | Teaching Feedback for This Week |

**Phase Two: Severe Respiratory Condition**

| **The Second Week** | Monday | Morning | Following up with doctors on rounds |
| --- | --- | --- | --- |
|  |  |  | Tracheal intubation fixation |
|  |  |  | Tracheostomy fixation |
|  |  |  | Case collection |
|  |  | Afternoon | Severe respiratory observation |
|  |  |  | Respiratory-related videos |
|  | Tuesday | Morning | Rounds with Doctors |
|  |  |  | Lung auscultation |
|  |  |  | Oral pharynx/nasopharyngeal airway use |
|  |  |  | Case collection |
|  |  | Afternoon | Writing case reports |
|  | Wednesday | Morning | Following up with doctors on rounds |
|  |  |  | Tracheal extubation |
|  |  |  | Ventilator weaning process |
|  |  |  | Artificial airway suctioning |
|  |  | Afternoon | Writing case reports |
|  | Thursday | Morning | Following up with doctors on rounds |
|  |  |  | Intra-cuff pressure measurement |
|  |  |  | ETCO_2_ monitoring |
|  |  |  | Filter |
|  |  | Afternoon | Writing case reports |
|  | Friday | Morning | Following up with doctors on rounds |
|  |  |  | Oxygen and humidification devices |
|  |  |  | Filter |
|  |  | Afternoon | Writing case reports |
|  |  |  | Teaching feedback this week |

**Second Phase: Severe Respiratory Condition**

| **The Third Week** | Monday | Morning | Following up with doctors on rounds |
| --- | --- | --- | --- |
|  |  |  | Open and Closed Suctioning Techniques |
|  |  |  | Simple Breathing Device |
|  |  | Afternoon | Writing case reports |
|  | Tuesday | Morning | Following up with doctors on rounds |
|  |  |  | Ventilator Safety Check |
|  |  |  | Basic Ventilator Operation |
|  |  | Afternoon | Writing case reports |
|  | Wednesday | Morning | Following up with doctors on rounds |
|  |  |  | Analyzing Ventilator Parameters |
|  |  |  | Recording Ventilator Parameters |
|  |  | Afternoon | Writing case reports |
|  | Thursday | Morning | Following up with doctors on rounds |
|  |  |  | Ventilator Mode (I) |
|  |  | Afternoon | Writing case reports |
|  | Friday | Morning | Following up with doctors on rounds |
|  |  |  | Ventilator Mode (II) |
|  |  |  | Ventilator Alarms |
|  |  | Afternoon | Writing case reports |

**Second Phase: Severe Respiratory Condition**

| **The Fourth Week** | Monday | Morning | Following up with doctors on rounds |
| --- | --- | --- | --- |
|  |  |  | Breathing circuit replacement |
|  |  |  | Breathing circuit replacement |
|  |  | Afternoon | Writing case reports |
|  | Tuesday | Morning | Following up with doctors on rounds |
|  |  |  | Portable ventilator |
|  |  |  | Non-invasive ventilator |
|  |  |  | Oxygen cylinder |
|  |  | Afternoon | Group discussion (abnormalities of artificial airways) |
|  |  |  | Literature sharing (oxygen therapy) |
|  |  |  | Ventilator modes and parameters |
|  | Wednesday | Morning | Selection of excellent case reports |
|  |  |  | Teaching feedback meeting |
|  |  | Afternoon | Respiratory system assessment |
|  |  |  | Oxygen therapy (literature sharing) |
|  |  |  | Non-invasive positive pressure ventilation care |
|  | Thursday | Morning | Following up with doctors on rounds |
|  |  |  | Pulse Oxygen Saturation |
|  |  |  | Negative Pressure Aspiration Device |
|  |  |  | Fiberoptic Bronchoscopy Examination Nursing Cooperation Operation |
|  |  | Afternoon | Closed Thoracic Drainage (Group Standard) |
|  |  |  | Respiratory System Assessment |
|  |  |  | Difficult Extubation |
|  | Friday | Morning | ARDS Nursing Care |
|  |  |  | Prone Position Ventilation Nursing Care |
|  |  |  | Ventilator Transport |
|  |  | Afternoon | Phased Assessment and Analysis |

**Third Phase: Severe Hemodynamics**

| **The Fifth Week** | Monday | Morning | Following up with doctors on rounds |
| --- | --- | --- | --- |
|  |  |  | Bedside Monitor Operation |
|  |  |  | Portable Monitor Operation |
|  |  |  | ECG Lead Placement |
|  |  | Afternoon | Literature Sharing (Electrocardiogram monitor) |
|  | Tuesday | Morning | Following up with doctors on rounds |
|  |  |  | Non-invasive arterial blood pressure monitoring |
|  |  |  | Establishment of invasive arterial blood pressure monitoring |
|  |  |  | Arterial pressure waveform analysis |
|  |  | Afternoon | Introduction to hospitals and departments (for trainees) |
|  | Wednesday | Morning | Following up with doctors on rounds |
|  |  |  | 12-lead electrocardiogram |
|  |  |  | Identify common types of arrhythmias |
|  |  | Afternoon | Identify common types of arrhythmias |
|  |  |  | Theoretical knowledge of electrocardiograms |
|  |  |  | Sudden arrhythmia (group discussion) |
|  | Thursday | Morning | Following up with doctors on rounds |
|  |  |  | Arterial Catheter Blood Sampling Procedure |
|  |  |  | PICCO Monitoring |
|  |  | Afternoon | Literature Sharing |
|  |  |  | Hemodynamic Monitoring (Lecture) |
|  | Friday | Morning | Following up with doctors on rounds |
|  |  |  | Trainees Nursing Rounds |
|  |  | Afternoon | Ultrasound Workshop |

**Third Phase: Severe Hemodynamics**

| **The Sixth Week** | Monday | Morning | Following up with doctors on rounds |
| --- | --- | --- | --- |
|  |  |  | Trainees Nursing Rounds |
|  |  | Afternoon | Nursing Care for Hypotension Patients (Group Discussion) |
|  |  |  | Circulatory System Assessment and Monitoring |
|  | Tuesday | Morning | Following up with doctors on rounds |
|  |  |  | Micropump |
|  |  |  | Infusion Pump |
|  |  | Afternoon | Septic Shock Care |
|  |  |  | Septic Shock Care |
|  | Wednesday | Morning | ECMO Trainees Nursing Rounds |
|  |  |  | ECMO Nursing |
|  |  | Afternoon | ECMO Workshop |

**Fourth Phase：Severe Nutrition and Digestion**

| **The Sixth Week** | Thursday | Morning | Following up with doctors on rounds |
| --- | --- | --- | --- |
|  |  |  | Trainees Nursing Rounds |
|  |  |  | TPN Configuration |
|  |  |  | Nutritional Assessment |
|  |  | Afternoon | Trainees Teaching Training |
|  | Friday | Morning | Auscultation of Bowel Sounds |
|  |  |  | Tolerance to Enteral Nutrition |
|  |  |  | Enteral Nutrition Pump |
|  |  |  | Intra-abdominal Pressure Monitoring |
|  |  | Afternoon | Gastrointestinal Hemorrhage |
|  |  |  | Severe Pancreatitis |
|  |  |  | Management of Sudden Aspiration in Patients (Group Discussion) |
|  |  |  | Teaching Feedback |

**Fifth Phase：Severe Neurology and Rehabilitation**

| **The Seventh Week** | Monday | Morning | Following up with doctors on rounds |
| --- | --- | --- | --- |
|  |  |  | Head Position, Drainage Care |
|  |  |  | Intracranial Pressure Monitoring |
|  |  | Afternoon | Expert Lecture |
|  | Tuesday | Morning | Following up with doctors on rounds |
|  |  |  | Self-controlled analgesia pump |
|  |  |  | Trainees Nursing Rounds |
|  |  | Afternoon | Delirium Assessment/Care |
|  |  |  | ICU Syndrome Care |
|  |  |  | Pain and Sedation Score |
|  |  |  | Pain and Sedation Guidelines |
|  | Wednesday | Morning | Following up with doctors on rounds |
|  |  |  | Trainees Nursing Rounds |
|  |  |  | Trainees Nursing Rounds |
|  |  | Afternoon | Glasgow Score |
|  |  |  | Full Unresponsiveness Scale |
|  |  |  | Full Unresponsiveness Scale |
|  |  |  | Hypothermic Protection |
|  | Thursday | Morning | Following up with doctors on rounds |
|  |  |  | Trainees Nursing Rounds |
|  |  |  | Muscle Strength Assessment |
|  |  |  | Bed Mobility Assessment |
|  |  | Afternoon | ICU-Acquired Weakness |
|  |  |  | Early Mobilization of ICU Patient |
|  |  |  | Proper Limb Positioning for Critically Ill Patients |
|  | Friday | Morning | Trainees Nursing Rounds |
|  |  |  | Trainees Nursing Rounds |
|  |  | Afternoon | Reading Report Meeting |

**Six Phase：Severe Renal Disease**

| **The Eighth Week** | Monday | Morning | Operational Training |
| --- | --- | --- | --- |
|  |  | Afternoon | Operational Training |
|  | Tuesday | Morning | Operational Training |
|  |  | Afternoon | Operational Assessment |
|  | Wednesday | Morning | Following up with doctors on rounds |
|  |  |  | CRRT Nursing Rounds |
|  |  |  | Edema Assessment |
|  |  | Afternoon | Basic Theory of CRRT |
|  |  |  | CRRT Workshop |
|  | Thursday | Morning | Specialty ICU Visit |
|  |  | Afternoon | Teaching Feedback Session |
|  |  |  | Team Building |
|  | Friday | Morning | Graduation Ceremony |
